# Evaluating the Quality of Primary Transurethral Resection of Bladder Tumor: A Nine-Year Review at a Tertiary Healthcare Center

**DOI:** 10.7759/cureus.68143

**Published:** 2024-08-29

**Authors:** Muhammad Ali Khattak, Muhibullah Bangash, Wajahat Aziz, Sara Ghaffar, Ayesha Asghar, Yasir Iqbal, Habeeb Abdulrasheed, Awais Nawaz Khan, Asad Ali Khan

**Affiliations:** 1 Urology, University Hospitals Birmingham NHS Foundation Trust, Birmingham, GBR; 2 Urology, Aga Khan University Hospital, Karachi, PAK; 3 Emergency Medicine, Aga Khan University Hospital, Karachi, PAK; 4 Family Medicine, Aga Khan University Hospital, Karachi, PAK; 5 Acute and General Medicine, Queen Elizabeth Hospital Birmingham, Birmingham, GBR; 6 Internal Medicine, Ayub Medical College, Abbottabad, PAK; 7 Cardiology, University Hospitals Birmingham NHS Foundation Trust, Birmingham, GBR

**Keywords:** length of stay, body mass index, detrusor muscle, single instillation of chemotherapeutic agent, aga khan university hospital, european association of urology, transurethral resection of bladder tumor

## Abstract

Objective: This study aimed to assess the quality of primary transurethral resection of bladder tumor (TURBT) procedures performed at Aga Khan University Hospital (AKUH) over nine years, focusing on proper documentation, completeness of tumor resection, quality of histopathology reports, complication rates, and adherence to European Association of Urology (EAU) guidelines.

Materials and methods: A retrospective analysis of patients aged 25-75 who underwent primary TURBT at AKUH between 2010 and 2019 was done. Patients with incomplete records, concomitant procedures, or those who underwent emergency TURBT were excluded. Data was collected on patient demographics, clinical presentation, intraoperative details, and histopathology reports. Statistical analysis was performed using SPSS Version 27.0.

Results: 300 patients were initially identified, with 265 meeting the inclusion criteria. The mean age was 61.5 years, with 83% being male. Complete tumor resection was achieved in 35% (n=92) of cases, while deep biopsy was taken in 85% (n=226). Detrusor muscle (DM), a marker of resection quality, was noted in 75% (n=200) of histopathology reports. However, documentation quality varied, with 54% (n=143) of cases lacking clear information on resection completeness. The administration of a single instillation of a chemotherapeutic agent (SICA) was recorded in 79% (n=210) of patients, and the 30-day postoperative complication rate was monitored.

Conclusion: The study highlights areas for improvement in the quality of TURBT procedures at AKUH, particularly in the documentation of resection completeness and adherence to established guidelines. Ensuring thorough resection and proper documentation is critical to optimizing patient outcomes and future management plans.

## Introduction

Bladder cancer ranks as the ninth most frequently diagnosed cancer globally. The highest incidence rates are observed in men in Southern and Western Europe and North America, as well as in certain countries in Northern Africa or Western Asia [1]. In Pakistan, bladder cancer constitutes 5.6% of all cancer cases, with an incidence rate of 8.9 cases per 100,000 individuals [2]. Transurethral resection of bladder tumor (TURBT) is the first line for diagnosing, staging, and treatment of visible bladder tumors. TURBT is a critical surgical procedure performed to diagnose and treat bladder cancer. As the initial and most definitive therapeutic intervention, the quality of TURBT significantly influences patient outcomes, including diagnostic accuracy, staging precision, and long-term survival rates. Given its importance, evaluating and ensuring the quality of TURBT procedures is paramount. Basic principles of TURBT include resecting all grossly visible tumors and providing adequate tissue specimens for histological diagnosis. Determination of tumor grade, stage, depth of invasion, and concomitant Carcinoma in situ (CIS) is dependent upon the quality of TURBT [3]. 

Components of a good quality TURBT according to the EAU guidelines include cystourethroscopy before resection, complete tumor resection, presence of detrusor muscle (DM) in the specimen, and additional biopsies if indicated. As this quality is judged by reporting the final histopathology specimen, coordination between the surgery and histopathology team is important, and this includes a bladder diagram, proper labeling of specimens, and submitting fractions in separate containers. This should be achieved while keeping the cost and complications to a minimum [4,5].

The surgeon should attempt to completely resect the tumor, which can be done through piecemeal technique or via the en-bloc method. Complete resection is essential to achieve a good prognosis bloc resection. It is practically useful in selected exophytic tumors. It ensures adequately resected specimens containing DM in almost 96-100% of cases. The presence of DM in the specimen is a surrogate marker of completeness of resection. Incomplete resection, without having any DM tissue will require the second TURBT within two to six weeks. This is not only costly but also increases the patient burden and morbidity [5,6].

It has been studied and observed that the absence of DM in the specimen is linked with an increased risk of residual pathology due to incomplete removal, early recurrence, and inadequate staging of the tumor [7]. In a study of 94 patients with bladder cancer, after TURBT a marginal resection was obtained around the entire area of resection. 24 had a tumor positive margin while 60 had tumor negative margins. The recurrence rate (83%) was much higher in tumor margin-positive patients compared to 57% of patients with tumor-free margins [8].

The significance of high-quality TURBT cannot be overstated, as inadequate resection or staging inaccuracies can lead to suboptimal treatment decisions and poorer prognoses. In a systematic review done by Akand et al., it was found that there are multiple quality control indicators (QCI) but the literature supports the use of complete resection, repeat resection, the presence of DM, and intravesical instillations [9]. By systematically analyzing the outcomes of primary TURBTs at AKUH, this study aims to identify areas of excellence and opportunities for improvement. The ultimate goal is to enhance surgical practices, optimize patient care, and contribute to the broader medical community’s understanding of effective bladder cancer management. This study aims to assess the quality of primary TURBT procedures performed at Aga Khan University Hospital (AKUH), considering various factors such as completeness of tumor resection, complication rates, and adherence to European Association of Urology (EAU) guidelines and then make a proper pathway for patients presenting to AKUH for management of primary TURBT.

## Materials and methods

We performed a retrospective study about the quality of TURBT in patients undergoing primary TURBT in our tertiary center, AKUH, Karachi, Pakistan. We included all patients aged 25-75 years who underwent elective primary TURBT at AKUH between 2010 and 2019 with complete records available. The sampling technique was non-probability, consecutive sampling. Patients who had missing records, concomitant other procedures at the same time, and who had emergency TURBT were excluded. To identify a suitable cohort, we generated an electronic list of all operations performed by all consultants at our center between 2010 and 2019. Within this list, we searched for the codes, TURBT and bladder cancer. A total of 300 cases were retrieved, of which 35 were excluded. Among the excluded cases, 15 patients had incomplete records and 20 patients did not have primary TURBT, they came for redo, and TURBT and primary surgery were done at another center. Operation notes were screened to ensure that the patients met the inclusion criteria.

Since this was a retrospective study that did not involve any changes to patients, it was registered as an audit, and approval was taken from the Ethical Review Committee (ERC). Data collection was started after the ethical review committee exemption on specifically designed proforma. This was a retrospective review of medical records of patients and histopathology reports of all those patients who underwent primary TURBT as per selection criteria. Data was collected on proforma on the patient’s demographics, and clinical presentation was reviewed from the surgery's initial assessment form. Intraoperative details were retrieved from operative notes. From the bladder diagram on operating notes, data regarding size, site, number, and others was retrieved.

Observations regarding the frequency and timing of administration of single instillation of chemotherapeutic agent (SICA) were also assessed. Thirty-day postop complications and re-admission rates were recorded according to the Clavien System from hospital records and mortality and morbidity meeting data review. Only the principal investigator and co-investigators collecting the data had access to the data. Patient confidentiality was maintained using a specific code for each patient. This study did not interfere with the course of treatment of patients. The data collected was only used for the purpose of this study.

Descriptive statistics were used to summarize the baseline characteristics of the study population. Continuous variables, such as age and length of stay (LOS), were reported as mean and standard deviation (SD); body mass index (BMI) was reported as median and interquartile range (IQR). Categorical variables, such as gender, and co-morbid conditions were reported as numbers and percentages. For analysis of operative characteristics, quality of documentation, and quality of the histopathological report, categorical variables such as ASA, anesthesia, complete resection, tumor, deep biopsy and additional biopsy containers, the technique of resection, bladder diagram, tumor type and grade, CIS, DM in the specimen and its invasion, lamina propria and invasion, and SICA were reported as numbers and percentages. Continuous variables such as operative time and tumor size were reported as mean and SD; tumor number was reported as median and interquartile range. For analysis of outcome data, continuous variables, such as duration of healing, were reported as median. Categorical variables, such as readmission, reason for readmission, complications, complication type, and second TURBT within six weeks were reported as numbers and percentages. All the data were entered and analyzed using Statistical Package for the Social Sciences (IBM SPSS Statistics for Windows, IBM Corp., Version 27.0).

## Results

The study included a total of 300 patients of which 265 patients were included, 15 had incomplete records, and 20 patients did not have primary TURBT. Of the 265 patients who had primary TURBT, the mean age was 61.5 years (±12.3). Of these, 83% (n=221) were male, and 17% (n=44) were female. The median BMI of the patients was 25.5, with an IQR of 15.9 to 48.9. The most common co-morbidity among the patients was hypertension, present in 71% (n=142). 39.5% (n=79) of patients had diabetes mellitus, and 18% (n=36) had ischemic heart disease. A smaller proportion of the study population, 13.5% (n=27), were fit and healthy with no co-morbidities. Other co-morbid conditions included asthma (4.5%,n=9), chronic obstructive pulmonary disease (COPD) (3.5%, n=7), chronic kidney disease (CKD) (2.5%,n=5), and dyslipidemia (2.5%, n=5). A minority of patients, 0.5% each (one patient per condition), had other conditions including hepatitis B, hepatitis C, arthritis, Parkinson's disease, epilepsy, hypothyroidism, or schizophrenia. The average length of hospital stay was 2.5 days, with an SD of 1.7 days. Regarding the ASA physical status classification, 9% (n=23) were classified as ASA I, 64% (n=170) as ASA II, 25% (n=67) as ASA III, and 2% (n=5) as ASA IV (Table 1).

**Table 1:** Baseline Demographics SD: standard deviation; IQR: interquartile range; BMI: body mass index; ASA: American Society of Anesthesiologists; COPD: chronic obstructive pulmonary disorder; CKD: chronic kidney disease; LOS, length of stay

Baseline Demographics		
Parameter	Number(s)	Percentage(s)
Age (mean +/- SD)	61.5	+/- 12.3
Gender		
Male	221	83%
Female	44	17%
BMI (median and IQR)	25.5	(15.9-48.9)
Co-morbid conditions		
Hypertension	142	71%
Diabetes mellitus	79	39.5%
Ischemic heart disease	36	18%
Fit and healthy (no co-morbid)	27	13.5%
Asthma	9	4.5%
COPD	7	3.5%
CKD	5	2.5%
Dyslipidemia	5	2.5%
Others (hepatitis B and C, arthritis, Parkinson, epilepsy, hypothyroidism, and schizophrenia)	1	0.5%
LOS (mean and SD)	2.5	+/-1.7
ASA		
I	23	9%
II	170	64%
III	67	25%
IV	5	2%

Among the 265 patients in the study, 68% (n=181) had general anesthesia, while 32% (n=84) had spinal anesthesia for the procedure. Complete tumor resection was achieved in 35% (n=92) but not in 11% (n=30). For 54% (n=143), whether complete resection was achieved was not recorded in operative notes. Biopsies were taken from 100% (n=265) of patients. Containers were labeled as tumor containers for all biopsies (100%) and sent separately. Deep biopsy was taken and sent in separate containers in 85% (n=226), while in 15% (n=39), deep biopsy was not taken and sent separately. Additional biopsies were taken in 14% (n=36), four of these were random, and 32 were from the prostate, prostatic urethra, and bladder neck. No additional biopsies were taken in 86% (n=229). The average time for the surgery was 50.1 minutes, with an SD of 26.6 minutes. For the type of resection, 74% (n=197) had a conventional resection, 13% (n=35) had en-bloc resection, and the type was not mentioned for 13% (n=33) (Table 2).

**Table 2:** Operative Characteristics SD: standard deviation

Operative Characteristics		
Parameter	Number(s)	Percentage(s)
Anesthesia		
General	181	68%
Spinal	84	32%
Complete resection		
Yes	92	35%
No	30	11%
Not mentioned	143	54%
Biopsies	265	100%
Tumor container		
Yes	265	100%
Deep biopsy container		
Yes	226	85%
No	39	15%
Additional biopsies		
Yes	36	14%
No	229	86%
Operative time (mean +/- SD)	50.1	+/- 26.6
Type of resection		
Conventional	197	74%
En-bloc	35	13%
Not mentioned	33	13%

Bladder diagrams were made in 95% (n=251) of operative notes, while they were missing in 5% (n=14). The average tumor size recorded was 4.49 cm, with an SD of +/- 3.45 cm. The median number of tumors was one, with an IQR of one to 10 tumors. Tumor location was documented in 94% (n=248)of cases but not in 6% (n=17) of operative notes. Regarding the technique of resection, the conventional (piecemeal) technique was used in 74% (n=197), the en-bloc resection technique was used in 13% (n=35), and the type of resection was not specified in 13% (n=33) of operative notes (Table 3).

**Table 3:** Quality of Documentation SD: standard deviation; IQR: interquartile range

Quality of Documentation		
Parameter	Number(s)	Percentage(s)
Bladder diagram		
Yes	251	95%
No	14	5%
Tumor size (mean /- SD)	4.49	+/- 3.45
Tumor number (median and IQR)	1	(1-10)
Tumor location		
Yes	248	94%
Not mentioned	17	6%
Type of resection (technique)		
Conventional	197	74%
En-bloc	35	13%
Not mentioned	33	13%

The type of tumor was mentioned in 97% (n=256) of the histopathology reports, with only 3% (n=9) missing this information. Among the reported tumor types, 95% (n=252) were transitional/urothelial carcinoma, 1% (n=4) were squamous cell carcinoma, 3% (n=6) were interstitial cystitis, and both malakoplakia and adenocarcinoma were identified in 0.38% (one patient each) of cases. Tumor staging was mentioned in only 9% (n=23) of the histopathology reports, while it was not included in 88% (n=236). For 3% (n=6), staging was not applicable. CIS was reported in 1% (n=2) of cases, and not mentioned in 97% (n=257) of histopathology reports. For 2% (n=6), CIS was not applicable as those were cases of interstitial cystitis. Tumor grading was documented in 92% (n=243) of the reports, with 6% (n=16) of reports missing this information. In 2% (n=6) of cases, grading was not applicable. Of those graded, 48% (n=128) were classified as high-grade tumors, 43% (n=116) as low-grade, and 6%(n=14) as PUNLMP. For another 3% (n=6), grading was not applicable, and in one report, the grade was not mentioned. The presence of DM in the specimen was noted in 75%(n=200) of histopathology reports. In 18% (n=47) of cases, no muscle was found, while 5% (n=12) had no comment on muscle presence. For 2% (n=6), this was not applicable. Muscle invasion was not seen in 58% (n=153) of cases and was found in 18% (n=47) of histopathology results. It was not mentioned in 22% (n=59), and in 2% (n=6), muscle invasion was not applicable as those were cases of interstitial cystitis. Lamina propria was mentioned in 86% (n=228) of the reports, absent in 12% (n=31), and not applicable in 2% (n=6). Among those where it was mentioned, lamina propria invasion was present in 54% (n=142) and absent in 38% (n=100). In 6% (n=17), the status of lamina propria invasion was not mentioned, and it was not applicable in 2% (n=6). Lymphovascular invasion was not present in 15% (n=40) of histopathology reports and was noted in 0.38% (n=1). However, it was not mentioned in 82% (n=218) of reports and was not applicable in 2% (n=6) (Table 4).

**Table 4:** Quality of Histopathology Report CIS: carcinoma in situ; PUNLMP: papillary urothelial neoplasm of low malignant potential; DM, detrusor muscle

Quality of Histopathology Report		
Parameter	Number(s)	Percentage(s)
Tumor type mentioned		
Yes	256	97%
No	9	3%
Tumor types		
Transitional/urothelial	252	95%
Squamous	4	1%
Interstitial cystitis	6	3%
Malakoplakia	1	0.38
Adenocarcinoma	1	0.38
Tumor stage mentioned		
Yes	23	9%
No	236	88%
Not applicable	6	3%
CIS		
Yes	2	1%
No	257	97%
Not applicable	6	2%
Tumor grade mentioned		
Yes	243	92%
No	16	6%
Not applicable	6	2%
Tumor grade		
High	128	48%
Low	116	43%
PUNLMP	14	6%
Not applicable	10	3%
Not mentioned	1	
DM in specimen		
Muscle found	200	75%
Not applicable	6	2%
No comment on muscle	12	5%
No muscle found	47	18%
Muscle invasion		
Not applicable	6	2%
No	153	58%
Not mentioned	59	22%
Yes	47	18%
Lamina propria mentioned		
Yes	228	86%
No	31	12%
Not applicable	6	2%
Lamina propria invasion		
Yes	142	54%
No	100	38%
Not mentioned	17	6%
Not applicable	6	2%
Lymphovascular invasion		
Not applicable	6	2%
No	40	15%
Not mentioned	218	82%
Yes	1	0.38%

SICA, mitomycin, was administered to 79% (n=210), while 21% (n=55) did not receive it. Regarding readmissions, 60% (n=157) were readmitted, with the most common reason being a relook TURBT, which accounted for 48% (n=125). Other reasons included radical cystectomy and ileal conduit formation (9%, n=26), acute kidney injury (AKI) (1%, n=2), hematuria (1%, n=2), and urinary retention (1%, n=2). Postoperative complications were recorded in 5% (n=14) of patients, with the most frequent being hematuria, occurring in 3.40% (n=9). Other complications included fever (1.13%, n=3 patients) and urinary tract infections (UTI) (0.75%, n=2). The majority of patients (95%, n=251) experienced no complications. The severity of complications was classified using the Clavien-Dindo system: four patients had grade I complications, seven had grade II, one had grade III, and two had grade IV complications. Additionally, 6% (n=17) of patients underwent a second TURBT procedure within six weeks postoperatively, while 94% (n=248) did not require a second procedure as seen in the post-op outcome (Table 5).

**Table 5:** Post-op Outcome SICA: single instillation of chemotherapeutic agent; TURBT: transurethral resection of bladder tumor; AKI: acute kidney injury; UTI: urinary tract infection

Post-op Outcome		
Parameter	Number(s)	Percentage(s)
SICA given		
Yes	210	79%
No	55	21%
Readmission		
Yes	157	60%
No	108	40%
Reason of readmission		
Relook TURBT	125	48%
AKI	2	1%
Radical cystectomy and ileal conduit formation	26	9%
Hematuria	2	1%
Urinary retention	2	1%
Complications		
Yes	14	5%
No	251	95%
Complication type		
Fever	3	1.13%
Hematuria	9	3.40%
UTI	2	0.75%
No complications	251	94.72%
Clavien Dindo		
I	4	0.02%
II	7	0.03%
III	1	0.004%
IV	2	0.008%
2nd TURBT within six weeks		
Yes	17	6%
No	248	94%

## Discussion

In this study of 265 patients who underwent primary TURBT, the mean age was 61.5 years, with a male predominance of 83% (n=221). The median BMI was 25.5, and hypertension was the most common co-morbidity (71%, n=142). General anesthesia was used in 68% (n=181) of cases, and complete tumor resection was achieved in 35% (n=92) of patients. The conventional resection technique was most frequently used in 74% (n=197) of cases, with bladder diagrams documented in 95% (n=251). Tumor characteristics were generally well-documented, with 97% (n=256) of histopathology reports mentioning tumor type, predominantly transitional/urothelial carcinoma (95%, n=252). However, tumor staging was noted in only 9% (n=23) of reports. DM presence was confirmed in 75% (n=200) of cases, and muscle invasion was found in 18%(n=47). SICA was given to 79% (n=210) of patients, and postoperative complications occurred in 5% (n=14) of patients, with hematuria being the most common. Overall, 60% (n=157) of patients were readmitted, mainly for relook TURBT (48%, n=125), while (6%, n=17) patients required a second TURBT within six weeks.

Accurate and complete documentation of operative notes is very important in general urology practice. The information in these notes is crucial for planning postoperative care and managing the disease in the future. This becomes even more important in cancer cases, where precise documentation is vital for coordination with multidisciplinary teams, ensuring optimal patient outcomes [10]. The Royal College of Surgeons (RCS) requires surgeons to keep records that are accurate, complete, easy to read, and made at the time of the procedure. Their "Good Surgical Practice" guidance suggests that operative notes should be clear and preferably typed. These notes should include details like the date and time, urgency of the procedure, names of the surgeon and anesthetist, the procedure performed, incision details, diagnosis and findings, any issues or complications, extra procedures, tissue samples, prostheses used, closure technique, estimated blood loss, decisions on antibiotic and DVT prophylaxis, and postoperative care instructions [11]. The European Association of Urology (EAU) recommends including a diagram in the documentation that details key tumor features, such as size, type, location, and number in TURBT cases [12]. Documentation of the location of bladder tumors offers several technical advantages, such as helping guide a repeat TURBT, especially if the initial procedure was done by a different surgeon. Additionally, it has important prognostic implications, particularly in cases of high-risk non-muscle-invasive bladder cancer (HR-NMIBC) [13].

The presence of DM in histopathological specimens is a critical quality indicator, reflecting the completeness of tumor resection. In a study by Mariappan et al., involving 356 patients, DM was identified in 241 cases (67%), with a corresponding recurrence rate of 23.1%. In contrast, among the 115 patients (32%) who had no DM, the recurrence rate was 85.7% [14]. In our study, DM was present in 75% (n=200) of the histopathological specimens, absent in 18% (n=47), and not commented on in 5% (n=12). Similarly, in a study conducted by Shoshany et al. involving 332 patients, DM was present in the histopathology specimens of 265 patients, with a recurrence rate of 22%. In contrast, among the 67 patients where DM was absent, the recurrence rate increased significantly to 48% [15].

In a retrospective study by Dyer et al. (2009-2013) involving 1580 patients who underwent TURBT followed by radical cystectomy, significant discrepancies were found between initial and final cancer staging, with 40-49% of patients being under-staged (the cancer was more advanced than initially thought) and 22-27% over-staged. These findings highlight the critical role of accurate histopathological reporting in patient management [16]. In our study, tumor stage was not mentioned in 88% (n=236) of reports. Tumor grade was mentioned in 92% (n=243) of reports, and comments on DM were made in 75% (n=200). Similarly, comments on lamina propria were made in 86% (n=228) of reports.

In the Scottish multicenter quality improvement program, Mitomycin after TURBT was given to 71% of patients; in our study, mitomycin (SICA) was given to 79% (n=210) of patients [17]. Bladder cancer is a complex disease, and despite advancements in treatment, survival rates have only modestly improved over the past 30 years. This is often attributed to the cancer's diverse nature, but differences in how it is treated might also play a role. For example, in NMIBC, initial surgeries are sometimes incomplete, requiring additional procedures, and guidelines are not always followed. To address these issues, unified approaches are required to improve adherence to guidelines. Quality indicators are also used to assess and improve healthcare standards [18].

This study aimed to assess the quality of primary TURBT at AKUH and then to make an appropriate pathway for primary TURBT patients so that a unified approach is made to manage bladder cancer patients as per EAU guidelines. This was a single-center retrospective study with a smaller sample size and lack of strict follow-up. There can also be an element of observer bias. By implementing a standardized pathway for primary TURBT at AKUH, in line with EAU guidelines, we can potentially improve the quality of care for bladder cancer patients. Future prospective studies are essential to evaluate the long-term effectiveness of these measures in reducing recurrence rates and improving survival outcomes in NMIBC patients.

## Conclusions

While the quality of TURBT procedures at AKUH demonstrates adherence to many critical aspects of bladder cancer management, there is a clear need for enhanced surgical and histopathological documentation. Improving these aspects will not only align with international guidelines but also potentially reduce the rates of recurrence and the need for re-intervention, ultimately improving patient outcomes. The findings from this study provide a basis for developing targeted interventions to elevate the quality of TURBT procedures, thereby optimizing the overall care of bladder cancer patients.

## References

[1] Antoni S, Ferlay J, Soerjomataram I, Znaor A, Jemal A, Bray F (2017). Bladder cancer incidence and mortality: a global overview and recent trends. Eur Urol.

[2] Bhurgri Y, Bhurgri A, Hassan SH, Zaidi SH, Rahim A, Sankaranarayanan R, Parkin DM (2000). Cancer incidence in Karachi, Pakistan: first results from Karachi cancer registry. Int J Cancer.

[3] Herr HW, Donat SM (2008). Quality control in transurethral resection of bladder tumours. BJU Int.

[4] Mostafid H, Brausi M (2012). Measuring and improving the quality of transurethral resection for bladder tumour (TURBT). BJU Int.

[5] Brausi M, Collette L, Kurth K (2002). EORTC genito-urinary tract cancer collaborative group variability in the recurrence rate at first follow-up cystoscopy after TUR in stage Ta T1 transitional cell carcinoma of the bladder: a combined analysis of seven EORTC studies. Eur Urol.

[6] Babjuk M, Böhle A, Burger M (2017). EAU guidelines on non-muscle-invasive urothelial carcinoma of the bladder: update 2016. Eur Urol.

[7] Akand M, Muilwijk T, Cornelissen J (2019). Development of a prospective data registry system for non-muscle-invasive bladder cancer patients incorporated in the electronic patient file system. Front Oncol.

[8] Jancke G, Rosell J, Jahnson S (2012). Residual tumour in the marginal resection after a complete transurethral resection is associated with local recurrence in Ta/T1 urinary bladder cancer. Scand J Urol Nephrol.

[9] Akand M, Muilwijk T, Raskin Y, De Vrieze M, Joniau S, Van Der Aa F (2019). Quality control indicators for transurethral resection of non-muscle-invasive bladder cancer. Clin Genitourin Cancer.

[10] Guerero DN, Bruce A, Vayalapra S, Menon V, El Hadi M, Khashaba S (2022). Improving the quality of transurethral resection of bladder tumour (TURBT) operative notes following the European Association of Urology guidelines: a completed audit loop study. Cureus.

[11] Cook N, Parwaiz H, Norris K, Hunter I (2017). Reaudit of the quality of operation note documentation using the Royal College of Surgeons of England (RCS Eng) good surgical practice guidelines (2014) over a three year period: 0839. Int J Surg.

[12] Babjuk M, Burger M, Zigeuner R (2013). EAU guidelines on non-muscle-invasive urothelial carcinoma of the bladder: update 2013. Eur Urol.

[13] Palou J, Sylvester RJ, Faba OR, Parada R, Peña JA, Algaba F, Villavicencio H (2012). Female gender and carcinoma in situ in the prostatic urethra are prognostic factors for recurrence, progression, and disease-specific mortality in T1G3 bladder cancer patients treated with bacillus Calmette-Guérin. Eur Urol.

[14] Mariappan P, Zachou A, Grigor KM (2010). Detrusor muscle in the first, apparently complete transurethral resection of bladder tumour specimen is a surrogate marker of resection quality, predicts risk of early recurrence, and is dependent on operator experience. Eur Urol.

[15] Shoshany O, Mano R, Margel D, Baniel J, Yossepowitch O (2014). Presence of detrusor muscle in bladder tumor specimens--predictors and effect on outcome as a measure of resection quality. Urol Oncol.

[16] Dyer T, Siemens DR, Nippak P, Meyer J, Booth CM (2021). Histology at transurethral resection of bladder tumor and radical cystectomy for bladder cancer: insights from population-based data. Can Urol Assoc J.

[17] Mariappan P, Johnston A, Padovani L (2020). Enhanced quality and effectiveness of transurethral resection of bladder tumour in non-muscle-invasive bladder cancer: a multicentre real-world experience from Scotland’s quality performance indicators programme Copy. Eur Urol.

[18] Donabedian A (2005). Evaluating the quality of medical care. Milbank Q.

